# Local Delivery of Cannabinoid-Loaded Microparticles Inhibits Tumor Growth in a Murine Xenograft Model of Glioblastoma Multiforme

**DOI:** 10.1371/journal.pone.0054795

**Published:** 2013-01-22

**Authors:** Dolores Hernán Pérez de la Ossa, Mar Lorente, Maria Esther Gil-Alegre, Sofía Torres, Elena García-Taboada, María del Rosario Aberturas, Jesús Molpeceres, Guillermo Velasco, Ana Isabel Torres-Suárez

**Affiliations:** 1 Department of Pharmacy and Pharmaceutical Technology, School of Pharmacy, Complutense University, Madrid, Spain; 2 Department of Biochemistry and Molecular Biology I, School of Biology, Complutense University, Madrid, Spain; 3 Instituto de Investigación Sanitaria del Hospital Clínico San Carlos, Madrid, Spain; 4 Department of Pharmacy and Pharmaceutical Technology, School of Pharmacy, Alcalá University, Madrid, Spain; 5 Instituto de Farmacia Industrial, Complutense University, Madrid, Spain; University of Oklahoma Health Sciences Center, United States of America

## Abstract

Cannabinoids, the active components of marijuana and their derivatives, are currently investigated due to their potential therapeutic application for the management of many different diseases, including cancer. Specifically, Δ^9^-Tetrahydrocannabinol (THC) and Cannabidiol (CBD) – the two major ingredients of marijuana – have been shown to inhibit tumor growth in a number of animal models of cancer, including glioma. Although there are several pharmaceutical preparations that permit the oral administration of THC or its analogue nabilone or the oromucosal delivery of a THC- and CBD-enriched cannabis extract, the systemic administration of cannabinoids has several limitations in part derived from the high lipophilicity exhibited by these compounds. In this work we analyzed CBD- and THC-loaded poly-ε-caprolactone microparticles as an alternative delivery system for long-term cannabinoid administration in a murine xenograft model of glioma. In vitro characterization of THC- and CBD-loaded microparticles showed that this method of microencapsulation facilitates a sustained release of the two cannabinoids for several days. Local administration of THC-, CBD- or a mixture (1∶1 w:w) of THC- and CBD-loaded microparticles every 5 days to mice bearing glioma xenografts reduced tumour growth with the same efficacy than a daily local administration of the equivalent amount of those cannabinoids in solution. Moreover, treatment with cannabinoid-loaded microparticles enhanced apoptosis and decreased cell proliferation and angiogenesis in these tumours. Our findings support that THC- and CBD-loaded microparticles could be used as an alternative method of cannabinoid delivery in anticancer therapies.

## Introduction

Δ^9^-Tetrahydrocannabinol (THC), the main active component of the hemp plant *Cannabis sativa* [1], exerts a wide variety of biological effects by mimicking endogenous substances – the endocannabinoids – that bind to and activate specific cannabinoid receptors [2]. So far, two G protein–coupled cannabinoid-specific receptors have been cloned and characterized from mammalian tissues: CB_1_, abundantly expressed in the brain and at many peripheral sites, and CB_2,_ expressed in the immune system and also present in some neuron subpopulations and glioma cells [2], [3]. One of the most active areas of research in the cannabinoid field is the study of the potential application of cannabinoids in the treatment of different pathologies [4], [5]. Among these therapeutic applications, cannabinoids are being investigated as anti-tumoral agents [6], [7]. Thus, cannabinoid administration curbs the growth of several types of tumor xenografts in rats and mice [6], [7] including gliomas [8]–[10]. Based on this preclinical evidence, a pilot clinical trial has been recently run to investigate the anti-tumor action of THC on recurrent gliomas [11]. The mechanism of THC anti-tumoral action relies on the ability of this compound to: (i) promote the apoptotic death of cancer cells (ii) to inhibit tumour angiogenesis and (iii) to reduce the migration of cancer cells [6].

Aside from THC, *C. sativa* produces approximately 70 other cannabinoids although, unlike THC, many of them exhibit little affinity for CB receptors [5], [12]. Of interest, at least one of these components, namely cannabinol (CBD), has been shown to reduce the growth of different types of tumor xenografts including gliomas [13]–[17]. Although the mechanism of CBD anti-tumoral action has not been completely clarified yet, it has been proposed that CBD-induced apoptosis relies on an increased production of reactive oxygen species (ROS) [13], a mechanism that seems to operate also in glioma cells [14], [15]. To note, co-administration of THC and CBD – an option that is being therapeutically explored also for other applcations [5], [12]; has been shown to promote cancer cell death and reduce the growth of glioma xenografts [18], [19].

One of the factors limiting the efficacy of anticancer treatments is the difficulty to reach effective concentration of antineoplasic agents at the tumour site. For example, the poor water solubility of certain anticancer agents such as paclitaxel or camptothecin hinders their application and complicates direct parenteral administration. In the case of cannabinoids, several pharmaceutical preparations have been developed and approved for cannabinoid administration including oral capsules of THC (Marinol®, Unimed Pharmaceuticals Inc.) and of its synthetic analogue nabilone (Cesamet®, Meda Pharmaceuticasl) and an oro-mucosal spray of standardized cannabis extract (Sativex®, GW Pharmaceuticals). These formulations have been approved for several clinical applications [5], [20]. Specifically, cannabinoids are well-known to exert palliative effects in cancer patients [5], [20]. The best-established use is the inhibition of chemotherapy-induced nausea and vomiting [5], [6] (Marinol® and Cesamet®). Cannabinoids also inhibit pain, and Sativex® has been already approved in Canada and is currently subject of large-scale Phase III clinical trials for managing cancer-associated pain. However, from the perspective of the utilization of cannabinoid-based medicines as antineoplastic agents, one of the issues that needs to be clarified is whether systemic administration of cannabinoids allows reaching effective concentrations of these highly lipid soluble agents [21] at the tumor site without enhancing undesired side affects [5], [6].

Local administration of polymeric implants for interstitial sustained release of anti-neoplasic agents allows enhancing the concentration of anticancer active substances in the proximity of the tumour [22]–[26] and could be an alternative strategy to systemic delivery at least for certain types of cancer. The aim of the present study was therefore to evaluate the antitumor efficacy of biodegradable polymeric microparticles allowing the controlled release of the phytocannabinoids THC and CBD. Our findings show that administration of cannabinoid-loaded microparticles reduces the growth of glioma xenografts supporting that this method of administration could be exploited for the design of cannabinoid-based anticancer treatments.

## Materials and Methods

### Ethics statement animal work

This study was carried out in strict accordance with the Spanish regulation for the care and use of laboratory animals. The protocol was approved by the committee on animal experimentation of Complutense University (Permits Number: CEA-1334; CEA-67/2012; CEA-75/2012). All surgery was performed under sodium pentobarbital anesthesia, and all efforts were made to minimize suffering.

### Materials

Δ^9^-tetrahidrocannabinol (THC) and cannabidiol (CBD) were from THC Pharm GmbH (Frankfurt, Germany), poly-ε-caprolactone (PCL) (Mw: 42,500), polyvinyl alcohol (PVA, MW = 30,000–70,000) and Sigmacote® were from Sigma-Aldrich (St. Louis, MO, USA). Methylene chloride (DCM) (HPLC grade) and dimethylsulfoxide (DMSO) were from Panreac (Barcelona, Spain). All chemicals and reagents were used as received. In order to avoid cannabinoid binding to labware, materials were pre-treated with Sigmacote®.

### Cannabinoid solution

For in vivo administration to mice, cannabinoid solutions were prepared at 1% (v/v) DMSO in 100 µL of PBS supplemented with 5 mg/mL of bovine serum albumin. No significant influence of the vehicle was observed on any of the variables determined in this study.

### Microparticles preparation

Biodegradable polymeric microparticles (MPs) were prepared by the oil-in-water emulsion solvent evaporation technique. Briefly, 50 mg of drug and 500 mg of polymer were dissolved in 5 mL of methylene chloride. Subsequently, the organic solution was poured onto 250 mL of a 0.5% PVA aqueous solution under stirring at 3000 rpm for 6 min. The resulting O/W emulsion was then stirred for 3 h to evaporate the organic solvent. Finally, the resulting MPs were washed with distilled water, filtrated (0.45 µm membrane filters) and freeze-dried. Vitamin E acetate (5%) was added to the organic solution when preparing THC-loaded MPs in order to avoid THC oxidation. Blank MPs were prepared using the same procedure but without adding cannabinoids.

### Microparticles morphology and size distribution

Scanning electron microscopy (JSM 6400, Tokyo, Japan) was used to evaluate the shape and the surface morphology of the blank, CBD- or THC-loaded PCL MPs. Particle size distribution was analyzed using a Microtrac® SRA 150 Particle Size Analyzer (Leeds & Northrup Instruments, Ireland). Samples were prepared by resuspending 5 mg of MPs in distilled deionized water. The results correspond to microsphere diameter determined by percentage volume distribution.

### Analytical method

High performance liquid chromatography was used to quantify the cannabinoid loaded in the microspheres and the amount of cannabinoid released at different time-points. HP1050 series instrument (Hewlett Packard) using a Mediterranea®Sea C18 column (150*4.6 mm, 5 μm) (Teknokroma, Barcelona, Spain) equipped with a UV detector set at 228 nm was used. The isocratic elution was prepared with methanol:acetonitrile: water (52∶30∶18) adjusted to pH 4.5 with acetic acid as mobile phase at a flow rate of 1.8 mL/min.

### Drug content and encapsulation efficiency

Briefly, 10 mg of MPs were dissolved with 1 mL of methylene chloride. Subsequently, mobile phase was added to the solution in order to precipitate the polymer and extract the cannabinoid. Samples were filtered prior to analysis by HPLC.

The encapsulation efficiency was obtained by calculating the percent of total cannabinoid loaded in the microspheres, divided by the initial cannabinoid added during the preparation of the microspheres.

### In vitro release of CBD and THC from PCL microspheres

For the *in vitro* release studies, microspheres were incubated in PBS pH 7.4-Tween®80 0.1% (v/v) and maintained in a shaking incubator at 37°C (n = 3). At predetermined time intervals supernatants were withdrawn and media was replaced. The concentration of CBD or THC in the release medium was quantified by HPLC. The percentage of drug released was presented as a cumulative curve.

### Cell culture

U87MG human glioma cells were obtained from ATCC. Cells were cultured in DMEM containing 10% FBS and maintained at 37°C in a humidified atmosphere with 5% CO_2_.

### Nude Mouse Xenograft Model of Human Glioma

Tumors were generated in athymic nude mice (Harlan Laboratories). The animals were injected subcutaneously on the right flank with 5*10^6^ U87 human glioma cells in 0.1 ml of PBS supplemented with 0.1% glucose. Tumors were measured using an external caliper, every day of treatment, and volume was calculated by the formula: 4π/3 *(length/2) *(width/2)^2^. When tumors reached a volume of 200 mm^3^, mice were randomly distributed into 8 experimental groups and treated daily with vehicle of the corresponding cannabinoid in solution or with blank or cannabinoid-loaded MPs at a dose of 75 mg MPs every 5 days. Mice were monitored daily for health status and for tumor volumes. After 22 days of treatment mice were sacrified and tumors were removed, measured and weighted. The remaining microspheres were removed, freeze-dried and analyzed for drug content.

### Immunofluorescence from tumor samples

Samples from tumors xenografts were dissected and frozen. Sections (10 µm) were permeabilized, blocked to avoid nonspecific binding with 10% goat antiserum and 0.25% TritonX-100 in PBS for 90 min, and subsequently incubated with rabbit polyclonal anti-KI67 (1∶300; Neomarkers; 4°C, o/n), or mouse monoclonal anti-CD31 (1∶200; Cymbus Biotechnology LTD; 4°C, o/n) antibodies. Next, sections were washed and further incubated with the corresponding Alexa-594-conjugated secondary antibodies (Invitrogen; 90 min, room temperature). Nuclei were stained with Hoechst 33342 (Invitrogen; 10 min, room temperature) and mounted with Mowiol (Merck, Darmstadt, Germany). Fluorescence images were acquired using an Axiovert 135 microscope(Carl Zeiss, Thornwood, NY, USA).

### Terminal deoxyribonucleotidyl transferase–mediated dUTP nick end labeling

Terminal deoxyribonucleotidyl transferase–mediated dUTP nick end labeling (TUNEL) was done using the in situ cell death detection kit (Roche).

### Statistics

Statistical analysis for tumor volume data were performed by ANOVA with a *post hoc* analysis by the Student-Neuman-Keuls test.

## Results

### Preparation and characterization of cannabinoid-loaded microparticles

**Figure 1:**
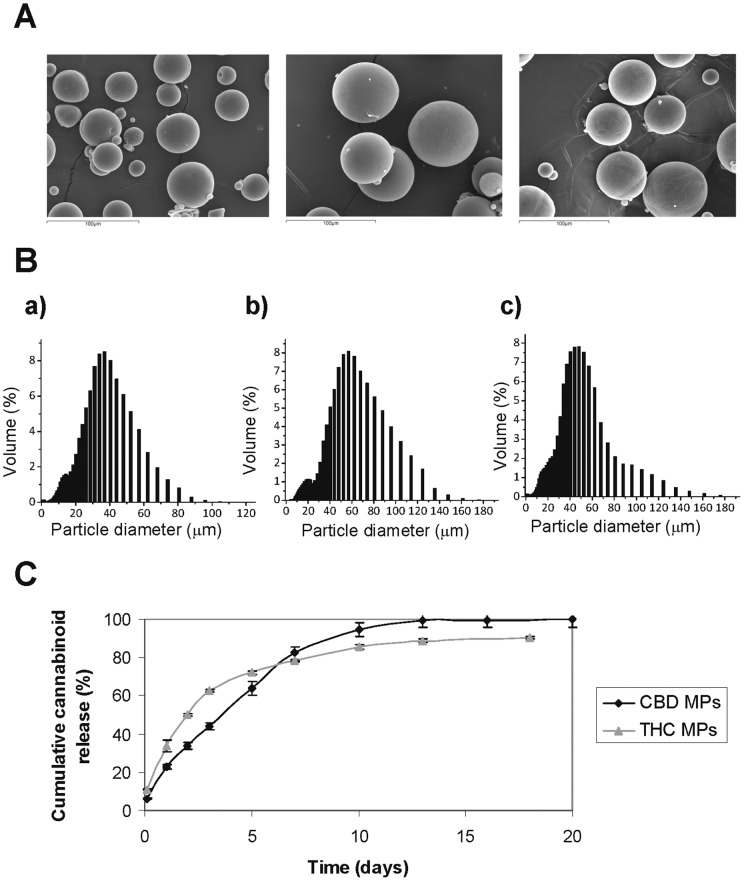
Characterization of cannabinoide-loaded microparticles. (A) Scanning electron microscopy (500X) of blank, CBD- and THC-loaded PCL MPs. Representative microphotographs of the three types of MPs are shown. (B) Particle size distribution of blank, CBD- and THC-loaded microspheres. Results correspond to microsphere diameter determined by percentage volume distribution. (C) Cannabinoid release profiles of THC and CBD-loaded PCL microspheres. For the in vitro release studies, microspheres were incubated in PBS pH 7.4-Tween®80 0.1% (v/v) and maintained in a shaking incubator at 37°C. At predetermined time intervals supernatants were withdrawn and media was replaced. The concentration of CBD or THC in the release medium was quantified by HPLC. Data correspond to the cumulative amount of each cannabinoid released at the indicated time points, and are expressed as mean percentage of released cannabinoid relative the total amount of cannabinoid loaded into the microspheres ± s.d (n = 3).

In order to evaluate the potential anticancer efficacy of microencapsulated cannabinoids, we prepared biodegradable polymeric poly-ε-caprolactone (PCL) microparticles (MPs) containing THC or CBD by using the oil-in-water emulsion solvent evaporation technique. Microparticles prepared by this procedure were spherical, showed a smooth surface (Figure 1A) and had an average size of 50 μm (Figure 1B). The encapsulation efficiencies of CBD and THC into PCL MPs were 99.09±5.14% and 84.55±13.6%, respectively. The release profile for the two types of MPs was characterized by a continuous release of CBD or THC for 13 days including a five-day initial burst release-phase during which 64% and 79% respectively of the total CBD or THC present in the MPs was released (Figure 1C and Table 1).

**Table 1:** In vitro analysis of the amount of CBD or THC released from cannabinoid-loaded microparticles.

Time (days)	mg CBD	mg THC
1	1.55	2.99
2	2.27	3.39
3	2.94	4.24
5	4.28	4.87
7	5.51	5.28
10	6.34	5.78
13	6.66	6.00
16	6.68	6.11
20	6.70	6.16

Microspheres were incubated in PBS pH 7.4-Tween®80 0.1% (v/v) and maintained in a shaking incubator at 37°C. At predetermined time intervals supernatants were withdrawn and media was replaced. The concentration of CBD or THC in the release medium was quantified by HPLC. Results correspond to the cumulative amounts of cannabinoid released in vitro from 75 mg MP.

### Evaluation of the anticancer activity of cannabinoid-loaded microparticles

**Figure 2:**
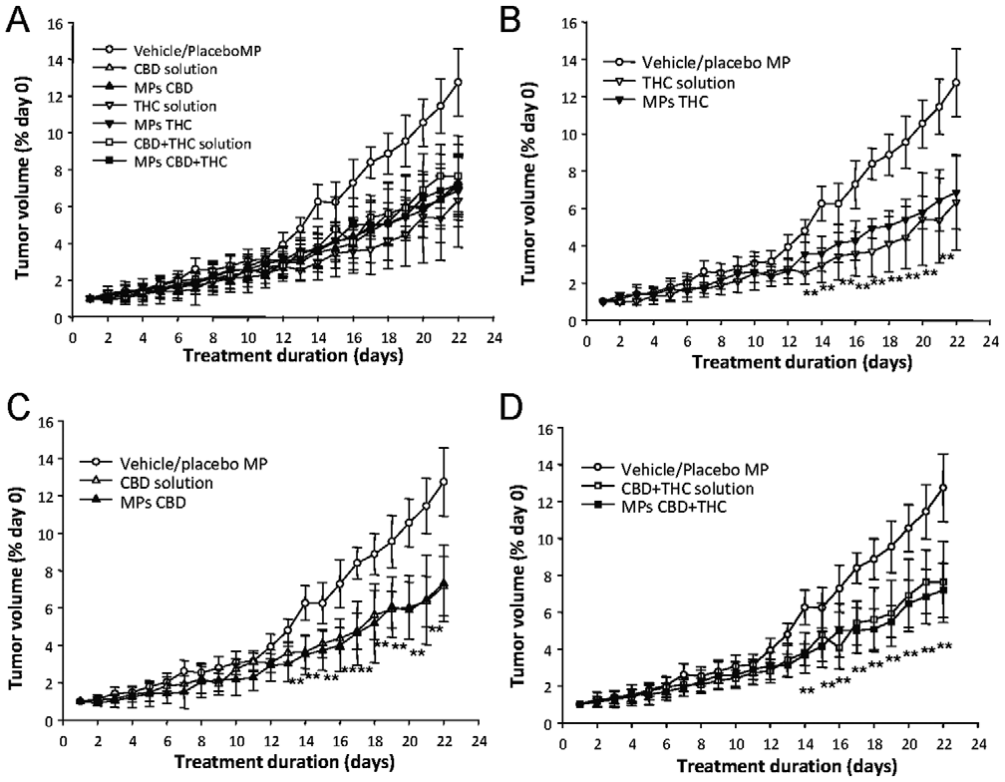
Cannabinoid-loaded microparticles reduce the growth of U87MG cell-derived tumour xenografts. (A) Effect of the local administration of placebo MPs, THC-loaded MP (75 mg of MP containing approximately 6.15 mg of THC per administration, one administration every 5 days), CBD-loaded MP (75 mg of MP containing approximately 6.7 mg of CBD per administration, one administration every 5 days), a mixture (1∶1 w:w) of THC- and CBD-loaded MP (37.5 mg of THC-loaded MP and 37.5 mg of CBD-loaded MP per administration, one administration every 5 days), THC (15 mg/kg/day corresponding to 0.5 mg THC per day), CBD (15 mg/kg/day corresponding to 0.5 mg THC per day) or THC + CBD (7.5 mg/kg/day of THC and 7.5 mg/kg/day CBD corresponding to 0.25 mg of THC and 0.25 mg of CBD per day) on the growth of U87MG cell-derived tumor xenografts. No significant differences were found between tumours treated with vehicle in solution or placebo MPs and these data were represented together. For the sake of clarity, comparisons between the effect of THC-loaded MPs and THC in solution (B), CBD-loaded MPs and CBD in solution (C), and THC-loaded MPs + CBD-loaded MPs and THC + CBD in solution (D) on the growth of U87MG cell-derived tumour xenografts are shown. Results are expressed as the mean fold increase ± SEM relative to vehicle treated tumors on the day one of the treatment. (n = 7). Tumours treated with THC-loaded MPs, CBD loaded MPs, a mixture of THC-loaded MPs and CBD-loaded MPs were significantly different (** p<0.01) from vehicle/placebo MPs-treated tumours. Tumours treated with THC in solution, CBD in solution or a mixture of THC and CBD in solution were also significantly different (p<0.01) from vehicle/placebo-treated tumours from day 14 until the end of the treatment (signs of significance are omitted for clarity). No significant differences were found among any of the treatments with cannabinoid-loaded microparticles and any of the treatments with cannabinoids in solution.

**Figure 3:**
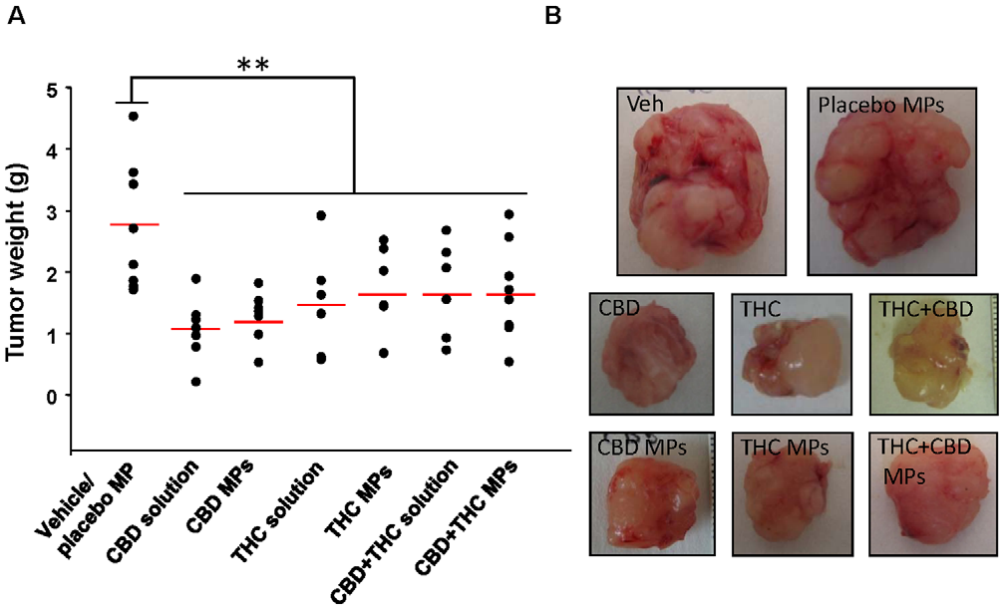
Cannabinoid-loaded microparticles reduce the weight of U87MG cell-derived tumour xenografts. (A) Effect of the local administration of placebo MPs, THC-loaded MP (75 mg of MP containing approximately 6.15 mg of THC per administration, one administration every 5 days), CBD-loaded MP (75 mg of MP containing approximately 6.7 mg of CBD per administration, one administration every 5 days), a mixture (1∶1 w:w) of THC- and CBD-loaded MP (37.5 mg of THC-loaded MP and 37.5 mg of CBD-loaded MP per administration, one administration every 5 days), THC (15 mg/kg/day corresponding to 0.5 mg THC per day), CBD (15 mg/kg/day corresponding to 0.5 mg THC per day) or THC + CBD (7.5 mg/kg/day of THC and 7.5 mg/kg/day CBD corresponding to 0.25 mg of THC and 0.25 mg of CBD per day) on tumour weight on the last day of the treatment. (B) Photographs of representative tumors of each experimental condition. (n = 7; ** p<0.01 from vehicle/placebo MPs-treated tumours).

To investigate the potential anticancer activity of the above-described cannabinoid-loaded MPs, we generated tumor xenografts by injecting subcutaneously U87MG cells (a well-established cellular model of glioma, that has been widely used to investigate the anticancer action of cannabinoids in this type of tumors [8], [10]) into the right flank of immnunodeficient mice. Once the tumours reached a 200–250 mm^3^ volume, animals were treated every 5 days with blank MPs (prepared in the absence of cannabinoids) or with microparticles loaded with THC or CBD. In addition, as the combined administration of submaximal doses of THC and CBD (1∶1) has been shown to reduce the growth of glioma xenografts [18], animal were also treated with a mixture of THC and CBD MPs.(1∶1 w:w) In the same experiment, another set of tumours was treated daily with a single peritumoral injection of a solution containing vehicle, THC, CBD or a mixture of THC and CBD (1∶1). As shown in Figure 2 administration every five days of cannabinoid-loaded microparticles (THC, CBD or THC + CBD) reduced tumor growth at the same extent than daily treatment with THC, CBD or THC + CBD in solution (Figure 2A–D). A similar effect was observed when the weight of the tumors on the last day of the treatment was analyzed (Figure 3A and 3B).

To note, animals treated with cannabinoids in solution and with cannabinoid-loaded MPs received approximately the same amount of cannabinoids along the treatment (Table 2). Thus, we found that 59 % of the initial amount of CBD present in CBD-loaded MPs (5.3±0.22 mg CBD/100 mg of MPs of the initial 8.93±0.13 mg CBD/100 mg MPs) and 58% of THC present in THC-loaded MPs (4.73±0.13 mg THC/100 mg MPs of the initial 8.21±0.07 mg THC/100 mg MPs) were still present in the MPs remaining at the site of injection at the end of the experiment (Table 2). Taken together, these observations support that administration of cannabinoid-loaded MPs every five days reduces tumor growth with the same efficiency than a daily injection of cannabinoids in solution and suggest that effective concentrations of cannabinoids could be reached at the tumour site using a lower frequency of MPs administration.

**Table 2:** Amount of THC or CBD administered to mice and released at the end of the treatment from cannabinoid-loaded microparticles.

	Cannabinoid-loaded MPs*	Cannabinoid-loaded MPs*	Cannabinoid-loaded MPs*	Cannabinoids in solution
	Total amount of cannabinoid administered (mg per animal)	Total amount of cannabinoid remaining on day 22 (mg per animal)	Estimated amount of cannabinoid released (mg per animal)	Total amount of cannabinoid administered (mg per animal)
**THC**	24.64 mg THC	14.19 mg THC	10.44 mg THC	10.5 mg THC
**CBD**	26.79 mg CBD	15.9 mg CBD	10.89 mg CBD	10.5 mg THC

* Animals received 75 mg of cannabinoid-loaded MPs every 5 days (corresponding to a total amount of 300 mg of microparticles per animal).

### Treatment with cannabinoid-loaded microparticles activates apoptosis and inhibits tumor angiogensis

**Figure 4:**
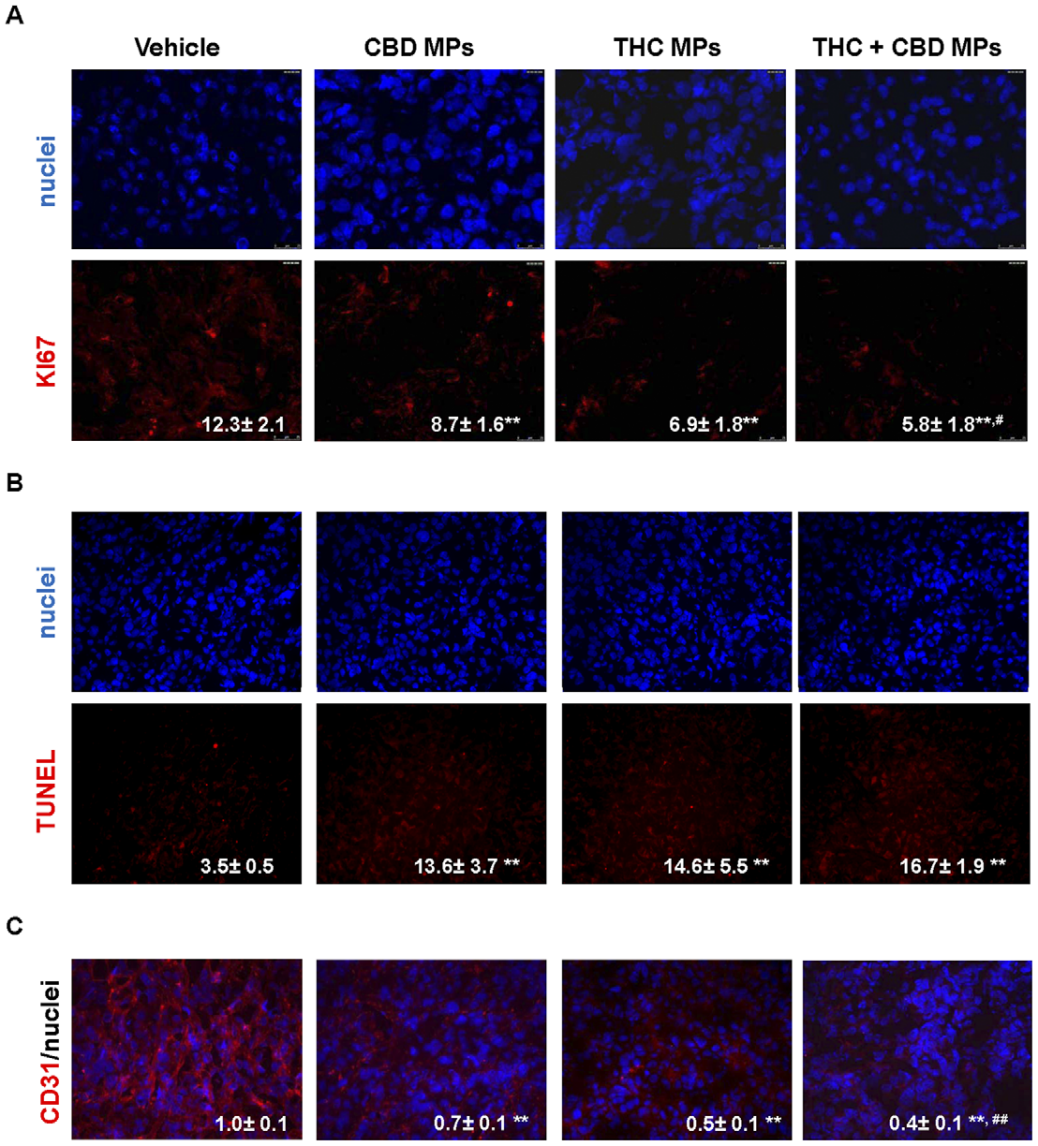
Cannabinoid loaded microparticles activate apoptosis and inhibit proliferation and angiogenesis of U87 MG cell-derived tumour xenografts. Effect of THC-loaded MP, CBD-loaded MP and a mixture of THC- and CBD-loaded MP on cell proliferation (as determined by KI67 immunostaining; A), apoptosis (as determined by TUNEL; B) and angiogeneis (as determined by CD31 immnunostaining; C) of U87MG cell-derived tumor xenografts. Values on the lower right corner of each panel correspond to the percentage of KI67-positive cells relative to the total number of nuclei in each section ± s.d. (A), the percentage of TUNEL-positive cells relative to the total number of nuclei in each section ± s.d. (B) or the CD31-stained area normalized to the total number of nuclei in each section (mean fold change ± s.d.; C) (10 sections of 3 different tumors from each condition were analyzed; ** p<0.01 from vehicle-treated tumors; ^#^ p<0.05 from CBD-loaded MP-treated tumors.

The mechanism of cannabinoid anticancer action relies on the ability of these compounds to promote cancer cell death – via stimulation of apoptosis – and inhibit cancer cell proliferation and tumour angiogenesis [6]. Therefore, we analyzed whether these mechanisms were activated in the tumour xenografts that had been treated with cannabinoid-loaded MPs. Unlike tumors that have been treated with blank MPs, treatment of U87-derived xenografts with THC- or CBD-loaded MPs or with a mixture of THC and CBD MPs reduced cancer cell proliferation (as determined by Ki67 immunostaing, Figure 4A), enhanced apoptosis (as determined by TUNEL; Figure 4B) and decreased tumour vascularization (as determined by immunostaining with the endothelial cell marker CD31, Figure 4C). These observations confirm that cannabinoid microencapsulation does not interfere with the mechanism by which these agents inhibit tumor growth.

## Discussion

One of the strategies that are currently under investigation to improve the efficacy of anticancer treatments is the utilization of drug carrier systems facilitating the local delivery of antineoplasic agents. Among these drug carrier systems, polymeric MPs have drawn much attention owing to their ability to control drug release, improve the therapeutic effect, prolong the biological activity, and decrease the administration frequency of several anti-neoplasic agents [27]–[29].

THC and CBD – two phytocannabinoids with potent anti-cancer activity – can be efficiently encapsulated into biodegradable PCL microspheres [30]. Our data show that PCL microspheres permit continuous release of these drugs and that its administration every 5 days to tumour-bearing mice reduces the growth of glioma xenografts with similar efficacy than a daily local administration of these cannabinoids in solution. Furthermore, results show that using this frequency of administration a significant fraction of the two cannabinoids is still present in the MPs at the end of the treatment. These observations suggest that effective concentrations of THC and CBD could be reached at the tumour site using a higher dosing interval.

Of note, different observations suggest that the doses of THC required to produce its cell death-promoting effect in cancer cells (IC 50 of around 1.5 to 6 μM in vitro depending on the type of cancer cell and the conditions of cell culture) are higher than the ones required for other actions of this agent or other CB_1_ receptor agonists in non-transformed cells [6]. Thus, reaching effective concentrations of THC at the tumour site using a systemic route of administration may require increasing the doses of THC administered to humans, which would enhance the risk of undergoing the undesired side effects of THC derived from its binding to CB1 receptors present in different brain regions. Local administration of cannabinoid-loaded MPs can help to circumvent this problem as their administration in the proximity of the tumour would ensure that effective concentrations of THC are reached at the therapeutically relevant site without enhancing acutely the levels of this agent in the brain regions responsible for its pyschoactivity. In addition, in this study we also found that the anticancer efficacy of the individual treatments with THC-loaded MP (containing approximately 6.15 mg of THC per administration) or CBD-loaded MP (containing approximately 6.7 mg of CBD per administration) is similar to that produced by co-administration of a mixture (1∶1 w:w) of THC- and CBD-loaded MPs (containing approximately 3.075 mg of THC and 3.75 mg of CBD per administration). These results are in line with previous observations by our laboratory [18], and suggest that rather than producing a synergistic effect, the combined administration of sub-maximal doses of THC and CBD could help to reduce the doses of these compounds required to produce their inhibitory effects on tumour growth.

Cannabinoids have been shown to produce a potent anticancer action in different types of tumour xenografts including some of the ones that exhibit a higher resistance to standard chemotherapies such as gliomas [8]–[10], pancreatic adenocarcinomas [31] and hepatocellular carcinomas [32], three tumour types that are susceptible of being treated with drug-loaded MPs [33]–[41]. This anticancer action of cannabinois is based on the ability of these compounds to enhance apoptosis, inhibit proliferation of cancer cells and inhibit tumour angiogenesis. Data presented here confirm that these mechanisms of action are activated in glioma xenografts upon administration of MPs loaded with THC, CBD or the combination of the two types of MPs. Although additional research should clarify whether a similar effect can be produced in other types of tumour xenografts, and whether MPs loaded with THC, CBD or its combination are equally efficacious in different tumour types and sub-types, these observations strongly support that microencapsulation could be a promising strategy to optimize the utilization of cannabinoids as anticancer agents.

Of interest, we have recently found that the combined administration of THC or THC + CBD [18] (but not CBD, S Torres, M Lorente and G Velasco unpublished observations) with temozolomide synergistically reduces the growth of glioma xenografts. The findings presented here now provide a rational for the design of novel anticancer strategies based on the use of cannabinoid-loaded MPs in combinational therapies.

## Conclusions

Data presented in this manuscript show for the first time that in vivo administration of microencapsulated cannabinoids efficiently reduces tumor growth thus providing a proof of concept for the utilization of this formulation in cannabinoid-based anti-cancer therapies.
